# Effect of Accelerated Ageing on the Mechanical and Structural Properties of the Material System Used in Protectors

**DOI:** 10.3390/polym11081263

**Published:** 2019-07-30

**Authors:** Longina Madej-Kiełbik, Katarzyna Kośla, Dorota Zielińska, Edyta Chmal-Fudali, Magdalena Maciejewska

**Affiliations:** 1 R&D Project Department, Institute of Security Technologies MORATEX, 3 M. Sklodowskiej-Curie Str., 90-505 Lodz, Poland; 2 Institute of Polymer and Dye Technology, Lodz University of Technology, 12/16 Stefanowskiego Str., 90-924 Lodz, Poland

**Keywords:** polymers, accelerated aging, impact protectors

## Abstract

Currently, there is a wide range of materials for motorcyclists available on the market that have a significant ability to absorb impact energy. Understanding the aging processes of materials is crucial for guaranteeing the long-term durability and safety of a new product. For this reason, the effect of accelerated aging on the mechanical and structural properties of the multifunctional materials used in commercial protectors was analyzed. The accelerated aging considered in this study simulated 3 years of use under real conditions. Then, DMTA and FT-IR research, as well as impact tests, were carried out on the commercially available protectors for motorcyclists, before and after the accelerated aging processes. Structural analysis using FT-IR showed no significant changes in the structure of the polymers used for producing the protectors. The DMA test results are consistent with those obtained from the impact study. Both methods showed that the samples maintain their protective properties, after accelerated aging. All of the examined protectors show that an increase in force is transferred through the sample, after the accelerated aging processes, but they still provide protection, according to the ISO standard.

## 1. Introduction

It is well-known that motorcycles are a very popular means of transportation and involve a relatively high risk associated with falls and collisions. This fact indicates the necessity of using special protective clothing, equipped with a shock-absorbing system, i.e., system of protectors, the task of which is to protect the rider against harm or injuries [1,2]. These materials provide protection in the event of a fall or an impact with other vehicles or objects. The shock absorbers must meet certain requirements relating to the appropriate protective clothing for motorcyclists, including an adequate resistance to impact, according to EN 1621-1:2012 Part 1—requirements and test methods for impact protectors. After many years of use, one should ensure that systems of materials and products, such as shock-absorbing protectors, have the required impact resistance and other mechanical properties, and that their aesthetic quality is not significantly diminished. Taking into the account the abovementioned information, from both a cognitive and practical point of view, gaining knowledge of the degradation of polymeric material systems and factors affecting the course of this process is an important issue. Among the undesirable environmental factors that may have a negative impact on the protectors used in clothing for motorcyclists, the temperature and various types of stresses should be identified. As a result of the degradation of materials, most of their properties may change. If so, those materials will not perform reliably in absorbing impact energy with the required performance. As is well-known, it should be ensured that shock-absorbing systems have the required strength and ability to absorb impact energy throughout their lifespan. Therefore, the selected protector systems were subjected to a simulation of accelerated aging in order to verify the effect of aging on their properties.

Motorbike clothing that provides protection against injury must, by law, be tested and marked as complying with the relevant standard. 

The first standard to be issued for motorcycle gear was for impact protectors, and it was released in 1997 (EN 1621-1). Standards have since been issued for gloves, boots, jackets, pants and back protectors. Each standard has a certain number, and the type of clothing that complies with it must have been tested and labelled with the CE label and the appropriate standard number.

The development of the standards has provided objective tests for measuring the protective performance of motorcycle clothing products. The tests are largely based on the research in [3], where a specification for motorcycle protective clothing and a definition of the injury risk and protection requirements for each part of the body were published.

Currently, a wide range of materials that are capable of absorbing a significant amount of impact energy and designed for the motorcyclists is available [4]. It should be ensured that material systems, especially shock-absorbing protectors, have the required impact resistance and mechanical properties and that their aesthetic merits are not significantly diminished [5,6,7,8]. During the use and the storage of these materials, their properties can deteriorate due to external aging factors, so it is important that the polymers are impact resistant in the long term [9]. Taking into the account the abovementioned information, from both a cognitive and practical point of view, gaining knowledge of the degradation of the tendencies of polymeric materials and factors affecting the course of this process is an important issue. 

Therefore, the selected protective systems were subjected to a simulation of accelerated aging, i.e., artificial aging, where the simulation of natural aging approximates, over a short period, the aging effects of long-term service conditions. The most widely used model for the lifetime prediction of materials is the Arrhenius model [10,11,12]. This study adopts the Arrhenius methodology for the accelerated aging of personal protectors. 

The personal protectors should not significantly change the impact properties over time or, if an alteration occurs, the structural properties. The level of reduction performance will be promoted, and this is the main objective of the present research. Therefore, the effect of accelerated aging on the physical and chemical parameters, as well as the energy absorption capabilities, of the tested material systems, both before and after the accelerated aging process, was studied. The aim of the research was to analyze the selected physical and chemical parameters, as well as the energy absorption capabilities, of the tested material systems, both before and after the accelerated aging process, and to understand the impact of the accelerated aging process on the mechanical and structural properties of the tested protectors. Consequently, the influence of aging was followed by a measurement of impact resistance, FTIR spectroscopy and DMTA. The problem of injuries among people practicing performance and recreation sports has been growing in recent years. Every year, tens of millions of people around the world sustain various kinds of sports injuries, with some of these injuries causing death or permanent disability [13]. For this reason, the protective properties of various types of sports protectors have attracted the interest of many societies linked with active sporting and the treatment of associated injuries and companies that offer a variety of shielding products. The issues presented in the publication attempt to answer questions related to possible changes in the protective properties of protectors designed for use in highly traumatic motorsports.

## 2. Materials and Methods

Commercially available protectors for the limbs and joints of motorcyclists that absorb impact energy were selected. The main constituent of the protectors in sample 1 and 2 is polyurethane, while for sample 3, it is ethylene-vinyl acetate copolymer (EVA). All samples showed a level 2 performance, according to the manufacturer. Samples had an irregular shape, as presented in Table 1.

### 2.1. Density Determination by the Hydrostatic Method

The density of solids, determined by the hydrostatic weighing method, are considered [15]. Hydrostatic density measurement consists of weighing an object of a known mass, while it is suspended from a balancing structure in a liquid with some assumed density value. The indicated loss of mass being equal to the mass of the displaced liquid, we are able to calculate the true volume of the unknown density. To precisely measure the mass of a weight in air, buoyancy correction of the air for the volume of the weight is required.

The densities of the examined objects were determined using the hydrostatic method and were calculated using Equation (1):

(1) $$\rho =\frac{A}{A-B}\cdot \rho_{0}$$

where ρ is the density of the sample [g/cm^3^], A is the weight of the sample in air [g], B is the weight of the sample in fluid [g], and ρ_0_ is the fluid density (0.9982 g/cm^3^, temperature of the density measurement: 20 °C).

### 2.2. Fourier Transform Infrared Study (FTIR)

Samples (both before and after the accelerated aging process, as shown in 3.5.) were characterized using an FTIR spectrophotometer (Thermo Scientific Nicolet iS10, Waltham, MA, USA). In order to perform IR analysis correctly, two measurements were conducted: the background spectrum of the crystal and the spectrum of the test sample. The FTIR spectrum was determined as the ratio of the sample spectra and the background spectrum (when performing the background spectrum, the response of the spectrometer itself was measured, without any sample). Dividing the spectrum of the sample by the background spectrum (which is called “rationing”) removes the adverse effects caused by the instrument and ambient conditions. Thus, the signals present in the final spectrum exclusively come from the sample.

The tests were performed using the single-reflection method, with the following device work parameter settings: DTGS KBr detector; measuring range: 4000–600 cm^−1^; accuracy of the measurement recording: 2; mirror speed: 0.31/s; aperture: 50; minimum number of recorded scans: 32; reflective snap of the ITR type (Thermo Scientific), with a diamond crystal in the reflection angle of 45°. Each scan was repeated three times and averaged.

### 2.3. DMTA Analysis

The tests using the DMA/SDTA 861e analyzer (Mettler Toledo, Greifensee, Switzerland), using stretching as the type of deformation, were performed. The samples were subjected to dynamic deformation, with a force of 1N, a deformation amplitude of 10.5 µm and a frequency of 1 Hz. The dimensions of the samples were 4 × 10.5 mm, and the thickness was about 3 mm. For each of the samples, measurements in the temperature range of −60 °C to 50 °C, with a heating rate of 3 °C/min were conducted. The modulus of elasticity E’ was determined, as well as the loss modulus E’’ and the mechanical loss factor (tan δ = E’’/E’), as a function of the temperature. The tests for the samples of polymeric materials, before and after the aging process, were performed.

### 2.4. Impact Resistance Study

**Figure 1:**
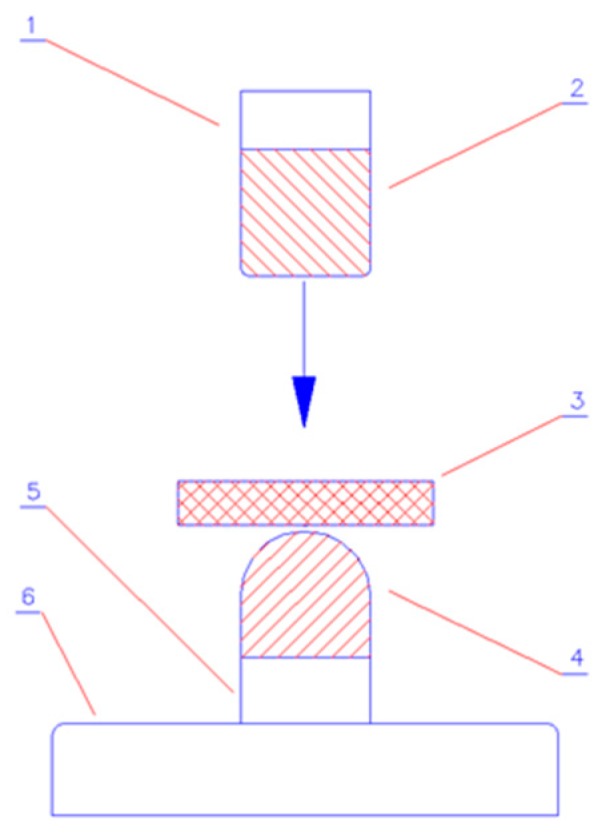
Design of a device for testing the shock-absorbing properties of the impact samples: 1—handle for fixing the hammer, 2—hammer releasing device, 3—sample, 4—anvil, 5—force sensor, and 6—stand base.

An impact resistance study was performed, according to the presumptions of the standard, PN-EN 1621-1:2012—“Motorcyclists’ protective clothing against mechanical impact” [16]. To perform the impact tests, a drop tower was used, which allows for a gravity drop of a mass along vertical guides, with an energy amount of (50 ± 2) J, onto a sample placed on a test anvil. A diagram of the construction of the drop tower is shown in Figure 1.

The principle of the device operation is as follows: along the vertical guides, a hammer of a specific mass falls, with a certain amount of energy, onto a sample placed on an anvil, which is attached to the base of the device. During the test, the force transmitted to the anvil, under the tested sample, is recorded by a piezoelectric force sensor, which is mounted on the anvil.

During the above-mentioned tests, a drop hammer made of polished steel, with a flat surface of 40 × 80 mm, rounded edges of 50 ± 5 mm and a mass of 5000 ± 10 g, was used. An anvil made of polished steel, with a semi-circular surface, a radius of 50 mm and a height of 180 ± 20 mm, was also used. The examination was performed under ambient conditions, and the samples were conditioned beforehand for 48.0 ± 0.5 h, at a temperature of 23 ± 2 °C and relative humidity of 50 ± 5%.

Regarding the impact attenuation, the standard, PN-EN 1621-1:2012 [16], introduces two levels of performance:

1. Level 1 is considered to be the required minimum for the sample to provide practical protection during an accident and ensures an optimal level of comfort in each riding position,
2. Level 2 provides an increased performance, but some inconvenience concerning weight and comfort is allowed.

The values of the transferred force for each level are stated in Table 2.

Based on the obtained data, it can be stated that each of the examined samples complies with level 2 performance, according to the standard, PN-EN 1621-1:2012.

### 2.5. Accelerated Ageing

Accelerated aging of the examined materials was performed using a thermostatic chamber SAL VIS LAB, type IC-80 (Renggli AG, Rotkreuz, Switzerland).

Simulation of the accelerated aging process using the Arrhenius formula, according to ASTM 1980F, was performed [17]:

(2) $$AAF=Q_{10}\left[\frac{T_{AA}-T_{RT}}{10}\right]$$

where AAF is the accelerated aging factor, T_AA_ is the accelerated aging temperature [°C], T_RT_ is the storage temperature in the real-time aging of the sample [°C], and Q_10_ is the aging factor, determined using the kinetics of changes in the selected property/parameter of the material, at temperature changes of 10 °C.

In order to calculate the real aging time, the following equation was used:

ATT = (365 days)/AAF(3)

where ATT is the time of accelerated aging, which is equivalent to the real-time aging (corresponding to 3 years of use under real conditions).

The parameter values of TAA = 65 °C, T_RT_ = 22 °C and Q_10_ = 2, as well as the corresponding period of 3 years of use under real conditions, were assumed [18,19,20]. The implementation of the above-listed values, shown in Formulas 2 and 3, included the following coefficient values: AAF = 19.70 and ATT = 56 days.

## 3. Results and Discussion

### 3.1. Density Analysis

The densities of the tested samples are shown in Table 3.

All samples have a similar level of density, regardless of whether the accelerated aging was performed or not. The most significant change for sample 1 was detected when the density increased by about 9%, after accelerated aging, in comparison to the initial sample of the tested material system. However, the detected change was insignificantly low in relation to the potential alteration to the performance relating to personal protection.

### 3.2. FTIR Analysis

**Figure 2:**
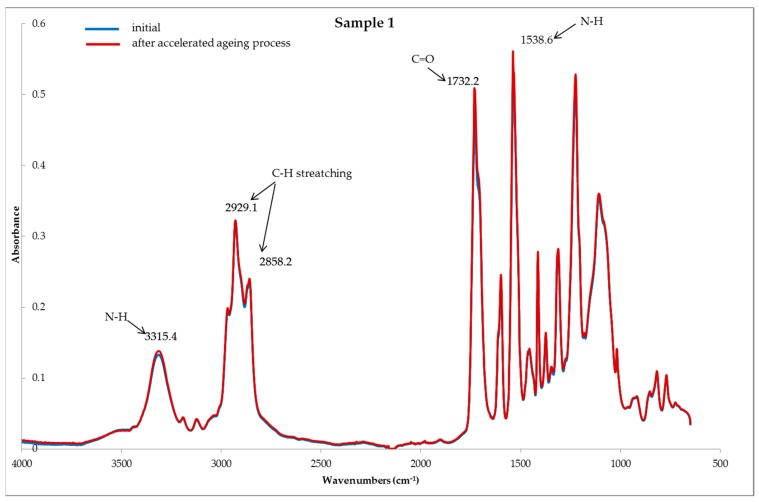
FTIR spectra of sample 1; samples, **---** initial (before) and **---** after the accelerated aging process.

**Figure 3:**
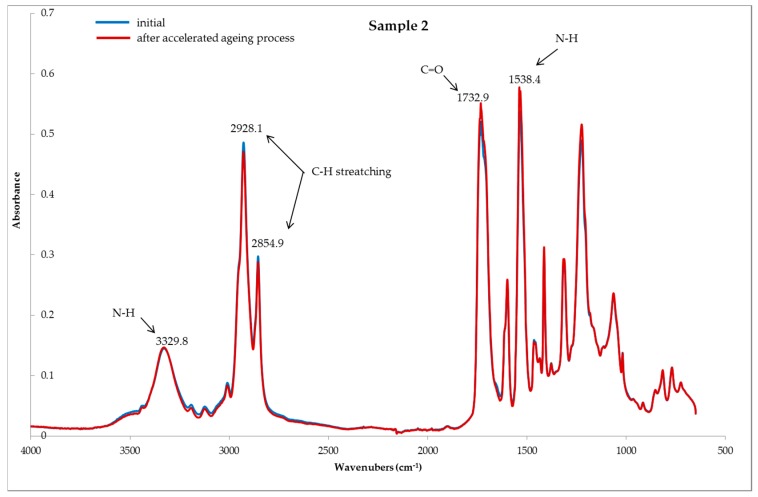
FTIR spectra of sample 2; samples, **---** initial (before) and **---** after the accelerated aging process.

**Figure 4:**
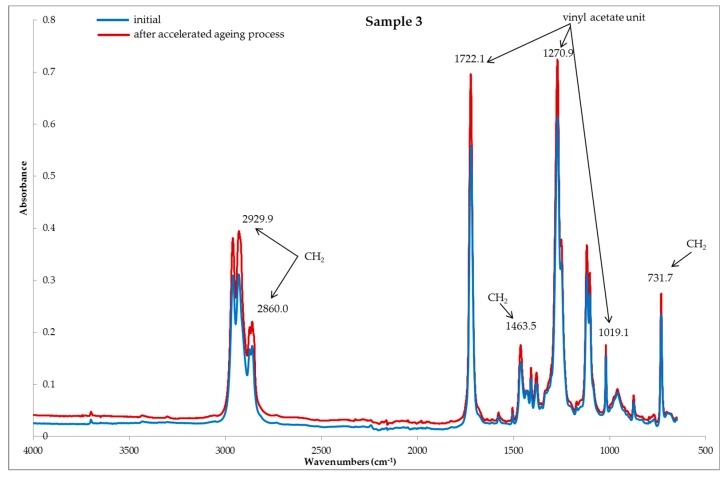
FTIR spectra of sample 3; samples, **---** initial (before) and **---** after the accelerated aging process.

Based on the FTIR spectra (Figure 2, Figure 3 and Figure 4) used for the studied systems, it was found that the accelerated aging process did not affect the structural and chemical changes of the polymers, i.e., the constituents, of the tested samples. This is evidenced by the lack of significant changes in the structure of the tested samples subjected to the process of accelerated aging, compared to the samples before aging.

Figure 2, Figure 3 and Figure 4 present the reflection FTIR spectra of the studied samples, before and after the process of accelerated aging.

The FTIR spectra of samples 1 and 2 are similar, and the main constituent of the protector systems is polyurethane. The characteristic absorption bands were found to be as follows: the absorption bands located in the range λ = 2850 cm^−1^–3000 cm^−1^ are indicative of the asymmetric and symmetric stretching vibrations of the methyl groups. The band located at λ = 3315 cm^−1^ is attributed to the stretching vibration of the N-H groups. The band at λ = 1533 cm^−1^ corresponds to the N-H deformation, whereas λ = 1728 cm^−1^ corresponds to the C=O groups [4].

The FTIR spectra of sample 3 suggested that the main constituent of the protector is ethylene-vinyl acetate copolymer (EVA). The characteristic absorbances of some bands assigned to the vinyl acetate units were identified at λ = 1721 cm^−1^, 1269 cm^−1^, 1019 cm^−1^, which may be related to the absorbance of the ethylene groups at λ = 2929 cm^−1^, 2860 cm^−1^, 1463 cm^−1^, and 731 cm^−1^.

### 3.3. DMTA Analysis

**Figure 5:**
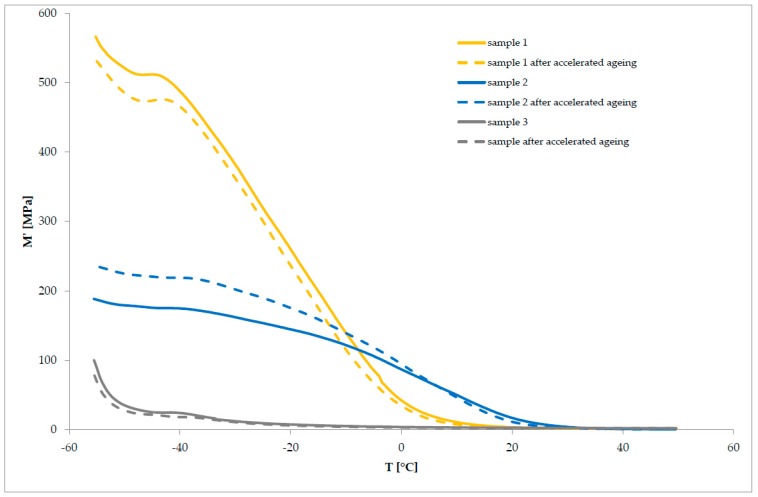
The storage modulus E’ in relation to the temperature of sample 1, sample 2, and sample 3, before and after the process of accelerated aging.

Subsequently, the characteristics of the studied polymeric material systems, before and after the accelerated aging process, were determined by DMTA. The storage modulus (Table 4) and the mechanical loss factor (Table 5), at temperatures of −20 °C and 20 °C, were determined for the samples, both before and after subjection to the accelerated aging process. Further, the cushioning properties of the examined materials were compared, and the impact of the accelerated aging process on these properties was determined. The samples of materials were tested, before and after the aging process. The obtained results are listed in Table 4 and Table 5 and in Figure 5.

The material systems under examination are characterized by different values of the storage module, both in the glassy and elastic state (Figure 5). The course of the change curves of the storage modulus, as a function of temperature, shows that sample 1 (E’_(−20 °C)_ approximately 262 MPa) exhibits the highest values of the storage modulus E’ under temperatures below 0 °C. These values, however, are significantly reduced as the temperature rises, only reaching 3 MPa at 20 °C. A decrease of the storage module value in this temperature range is accompanied by a broad peak on the mechanical loss factor curve, with a maximum of approximately tan δ = 0.65, which testifies to the relaxation process associated with the glassy/elastic state transition. After transitioning to the elastic state, sample 1 is characterized by the values of tan δ in the range of 0.65–0.20, so it shows good damping properties, although these are strongly dependent on temperature. Similar changes in dynamic properties are observed in sample 2 (Figure 5), which is characterized by a lower storage modulus and worse cushioning properties than sample 1 (E’_(−20 °C)_ approx. 145 MPa; tan δ _(−20 °C)_ approximately 0.09) at temperatures below −5 °C.

However, above this temperature, sample 2 exhibits a higher storage modulus than that of sample 1, which decreases with increasing temperature, reaching a value of approximately 17 MPa, at 20 °C, and below 1 MPa at the end of the measurement. At temperatures above −5 °C, the material’s ability to accumulate and dissipate energy during deformation, and thus its cushioning properties, are strongly dependent on the temperature. The value of the mechanical loss factor determined for sample 2 at 20 °C is comparable to that for sample 1.

Sample 3 exhibits the most dissimilar behavior under dynamic conditions (Figure 5).

Compared to the other samples, sample 3 is characterized by significantly low storage modulus values at negative temperatures (E’_(−20 °C)_ approximately 7 MPa), while it shows similar loss modulus values. This results in sample 3 having the highest mechanical loss factor among the tested samples, which is equal to the E”/E’ ratio (tan δ_(−20 °C)_ 0.42), at temperatures below zero. This proves that sample 3 has the best cushioning properties for this material within the discussed temperature range.

Moreover, the analysis of the storage modulus curve of sample 3 indicates that the modulus undergoes the smallest changes in a function of temperature among the examined samples, which means that the dynamic properties of the material are stable. However, the change in the mechanical loss factor, and its decrease with the increase of the measurement temperature to a value above 0.1, results from the change of the material’s ability to dissipate energy and indicates deterioration of the cushioning properties, along with a temperature increase within the range above zero.

**Figure 6:**
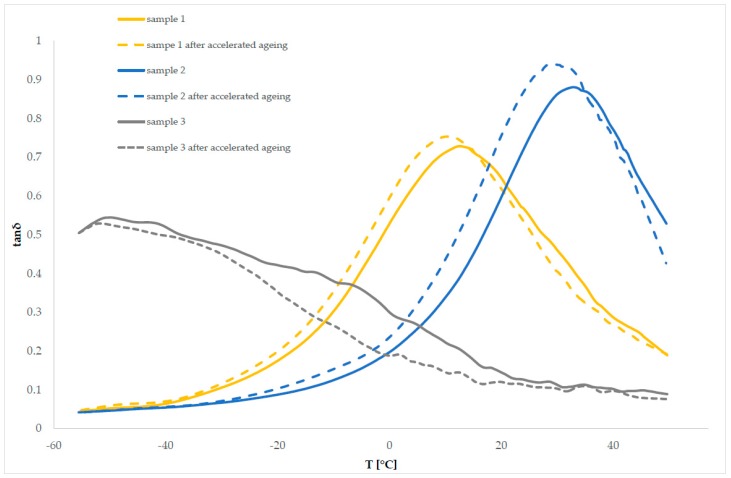
Mechanical loss factor tan δ in relation to the temperature for sample 1, sample 2, and sample 3, before and after the process of accelerated aging.

Comparing the curves of changes in the dynamic modules and the mechanical loss factor, as a function of temperature (Figure 6), as well as the determined values of the storage modulus and tan δ at temperatures of −20 °C and +20 °C, no significant effect of the aging process on the dynamic and cushioning properties of the tested samples was found. The values of E’_(−20 °C)_ were changed by several tens of MPa only for samples exhibiting high values of the storage modulus at negative temperatures (sample 1 and sample 2). However, the course of the curves of the loss modulus and tan δ had no significant impact on the cushioning properties of the examined polymer materials. Considering the range of changes in the values of tan δ and the accuracy of the measurement method, the process of aging did not cause significant changes in the cushioning properties of the material system under examination.

The tan δ values of sample 3 (Figure 6) were significantly lower, compared to the other samples. This indicates that this sample has weak/poor damping properties, which is confirmed by the impact study (the maximum force transferred under an examined sample was the highest for sample 3).

### 3.4. Impact Resistance Analysis

**Figure 7:**
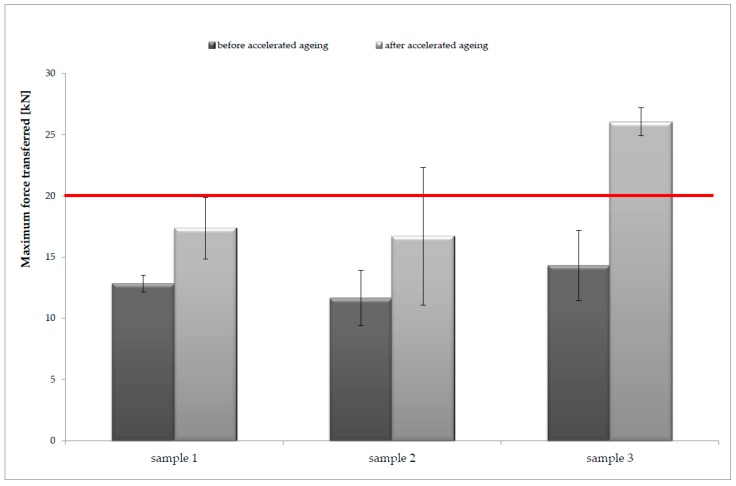
Dependence between the maximum force transmitted under the tested samples and the type of sample used for the tests, executed before and after the process of accelerated aging; the red line is the limit of the general mean value of force transferred for level 2 protection.

The lowest values of the maximum force transferred under the sample were obtained for sample 2: 11.65 ± 2.27 kN. The values for sample 1 and 3 are as follows: sample 1: 12.84 ± 0.68 kN; sample 3: 14.30 ± 2.89 kN. The results of the impact tests are presented in Figure 7.

After the accelerated aging process, an increase in the maximum value of the transmitted force was noted for each of the tested samples. Depending on the type of tested sample, there was an increase in the value of the transmitted force ranging from 18% to 45%. In the case of sample 3, the increase in the value of force transferred under the sample was so large that it caused a change in the level of protection from level 2, before accelerated aging, to level 1 after the accelerated aging process.

Sample 1 showed the best properties, i.e., the lowest value of the maximum force transmitted under the sample and the lowest range of changes (18%) in this parameter, after the accelerated aging process.

## 4. Conclusions

The obtained results of the research allowed the samples to be verified. The samples retained their properties (or the degree of change is acceptable) and functionality after the simulation of the accelerated aging processes. Shock absorbing systems, used in clothing for motorcycle riders, are an essential element for protecting users against harm or injuries. Based on the obtained research results concerning the examined systems, it can be concluded that

- For each of the protector system samples subjected to the accelerated aging process, an increase in the observed maximum value of the force transmitted under the sample was noted. This phenomenon may be due to the type of material used to make the protector system samples. Sample 1 showed the lowest value of the maximum force transmitted under the sample and the lowest range of changes (18%) in this parameter, after the accelerated aging process, compared to the other examined samples;
- The accelerated aging process caused a change in the level of protection from level 2, before accelerated aging, to level 1 after the accelerated aging process for sample 3. The other samples retained their protection level;
- The impact study, after the accelerated aging process, showed that, after the simulated 3 years, all samples should protect the limbs of motorcycle riders in the event of an accident;
- DMTA analysis showed that the accelerated aging process did not cause significant changes in the cushioning properties of the examined material systems; and
- The FTIR spectra did not show any significant structural and chemical changes in the polymers, confirming that there were few changes in the structure of the tested samples.

This research shows that the tested samples retain stable protective properties over time. We expect that the samples will protect the limbs of motorcycle riders in the event of an accident for 3 years, which will be validated in the next stage of the research. In the future, the samples should be exposed to natural weathering and climatic conditions for 3 years. After that, DMTA analysis and impact resistance analysis should be performed, and the results should be compared with the results obtained from the accelerated aging simulation.

## Figures and Tables

**Table 1:** Selected protectors used in clothing for motorcyclists.

Sample	View	Weight (g)	Length (cm)
		PBM-15/ITB:2006 [14]	
Sample 1	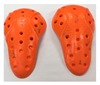	116.48 ± 0.48	22.5 ± 0.5
Sample 2	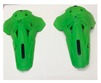	99.05 ± 0.01	27.8 ± 0.5
Sample 3	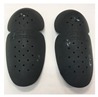	136.42 ± 1.28	19.9 ± 0.5

**Table 2:** Transferred force and performance levels [16].

	Level 1	Level 2
General mean value Single impact with area A * Single impact with areas B or C *	≤35 kN ≤35 kN <50 kN	≤20 kN ≤20 kN <30 kN

* A, B, C correspond to the areas indicated in Section 6.3.1.5.3 of the standard, PN-EN 1621-1:2012.

**Table 3:** Density of samples.

Sample	Density [g/cm^3^]	
	Before Accelerated Ageing (Initial Sample)	After Accelerated Ageing
Sample 1	0.5002 ± 0.0088	0.5478 ± 0.0096
Sample 2	0.3362 ± 0.0082	0.3403 ± 0.0059
Sample 3	0.7085 ± 0.0090	0.7126 ± 0.0125

**Table 4:** Storage modulus (of elasticity) of the material system samples.

Sample Code	Before the Process of Accelerated Aging		After the Process of Accelerated Aging	
	E’_(−20 °C)_ [MPa]	E’ _(20 °C)_ [MPa]	E’_(−20 °C)_ [MPa]	E’ _(20 °C)_ [MPa]
Sample 1	261.7 ± 4.5	3.2 ± 0.2	239.4 ± 4.4	2.6 ± 0.1
Sample 2	144.8 ± 6.7	16.6 ± 2.6	175.5 ± 3.4	10.6 ± 0.6
Sample 3	7.4 ± 0.2	2.6 ± 0.1	5.9 ± 0.3	2.2 ± 0.2

**Table 5:** Mechanical loss factor (tan δ) of the material system samples.

Sample Code	Before the Process of Accelerated Ageing		After the Process of Accelerated Ageing	
	tan δ_(−20 °C)_ [-]	tan δ _(20 °C)_ [-]	tan δ_(−20 °C)_ [-]	tan δ _(20 °C)_ [-]
Sample 1	0.17 ± 0.01	0.64 ± 0.03	0.19 ± 0.01	0.62 ± 0.02
Sample 2	0.09 ± 0.01	0.60 ± 0.04	0.10 ± 0.03	0.75 ± 0.02
Sample 3	0.42 ± 0.05	0.14 ± 0.02	0.35 ± 0.07	0.12 ± 0.02
